# Clinical and Biochemical Improvement After Switching From Agalsidase Alfa to Beta in a Boy With Classic Fabry Disease: A Case Report

**DOI:** 10.1002/ccr3.73353

**Published:** 2026-08-18

**Authors:** Nobuhiko Koga, Hiroaki Yodogawa, Takaaki Sawada, Yuichi Mushimoto, Shinichiro Nagamitsu, Kimitoshi Nakamura, Shinichi Hirose, Takahito Inoue

**Affiliations:** ^1^ Department of Pediatrics, School of Medicine Fukuoka University Fukuoka Japan; ^2^ Department of Pediatrics Kumamoto University Hospital Kumamoto Japan; ^3^ Department of Pediatrics, Faculty of Life Sciences Kumamoto University Kumamoto Japan; ^4^ Department of Pediatrics, Graduate School of Medical Sciences Kyushu University Fukuoka Japan; ^5^ General Medical Research Center, School of Medicine Fukuoka University Fukuoka Japan; ^6^ Center for Maternal, Fetal and Neonatal Medicine Fukuoka University Hospital Fukuoka Japan

**Keywords:** biomarker, drug antibody, enzyme, fabry disease, lysosome

## Abstract

We described a 12‐year‐old boy with classic Fabry disease who was diagnosed through newborn screening. At age 6.2, he started agalsidase alfa based on evidence of subclinical organ involvement. At age 7.2, acroparesthesia subsequently developed. At age 10.9, after switching to agalsidase beta due to an insufficient clinical and biochemical response, his acroparesthesia resolved and plasma Lyso‐Gb3 decreased. This report broadens the clinical understanding of children with Fabry disease.

**Key Clinical Message**

This is the first documented case of a male patient with classic Fabry disease who underwent a change in enzyme replacement therapy from agalsidase alfa to beta because of his clinical symptoms and Lyso‐Gb3 levels. The outcome of this study highlights the importance of monitoring symptoms and biomarkers in Fabry disease.

## 1 Introduction

Fabry disease (FD: MIM 301500) is an X‐linked lysosomal disorder caused by pathogenic variants in the *GLA* gene, resulting in a deficiency of α‐galactosidase A enzyme activity [1]. Reduced enzyme activity leads to accumulation of globotriaosylceramide, predominantly within the lysosomes of cells in multiple tissues, which can cause progressive damage to various organs [1, 2, 3, 4]. During childhood, male patients with very low α‐galactosidase A activity, known as the classic type, experience acroparesthesia (episodic neuropathic pain of the hands and feet), hypohidrosis, angiokeratoma, and gastrointestinal symptoms, which affect their quality of life [5, 6]. Acroparesthesia has persisted in many classic men throughout their lives [7]. Furthermore, untreated adult males with classic FD are at risk of developing renal failure, cardiac involvement, and severe vascular complications; among these, cardiac disease is a major determinant of prognosis, including the risk of arrhythmia and sudden death [1, 8, 9, 10].

Enzyme replacement therapy (ERT) with recombinant agalsidase has been available for the treatment of FD since 2001. There are two types of agalsidase: Alfa and beta, each with a different dosage [11]. In approximately half of adult male patients, acroparesthesia can be relieved with lower‐dose agalsidase alfa [12]. However, the appropriate enzyme selection for pediatric classical FD cases remains unclear. Furthermore, although ERT has improved outcomes for patients with FD, advanced FD‐related organ failure has been reported as irreversible [13, 14]. This poses a major challenge in determining FD cases as early as possible. To date, some countries have endeavored to diagnose FD as part of neonatal screening programs [15]. In some Japanese regions as well, newborn screening using measurement of α‐galactosidase A activity in white blood cells has been implemented, leading to an increase in the number of pediatric FD cases [16, 17]. However, the pediatric patient cohort remains limited, and information on the clinical course and treatment outcome is insufficient. Enzyme switching has been reported after the 2009 agalsidase beta shortage, including a 14‐year‐old boy who was switched from beta to alfa [18]. The only report of pediatric switching in the reverse direction involved a pre‐planned protocol evaluated primarily through renal biopsy [19]. Here, we report a Japanese boy with FD who was diagnosed through newborn screening. He switched from agalsidase alfa to beta because agalsidase alfa appeared clinically ineffective. To our knowledge, this is the first pediatric case documenting the clinical and biochemical course of a switch driven by insufficient efficacy.

## 2 Case History

**FIGURE 1:**
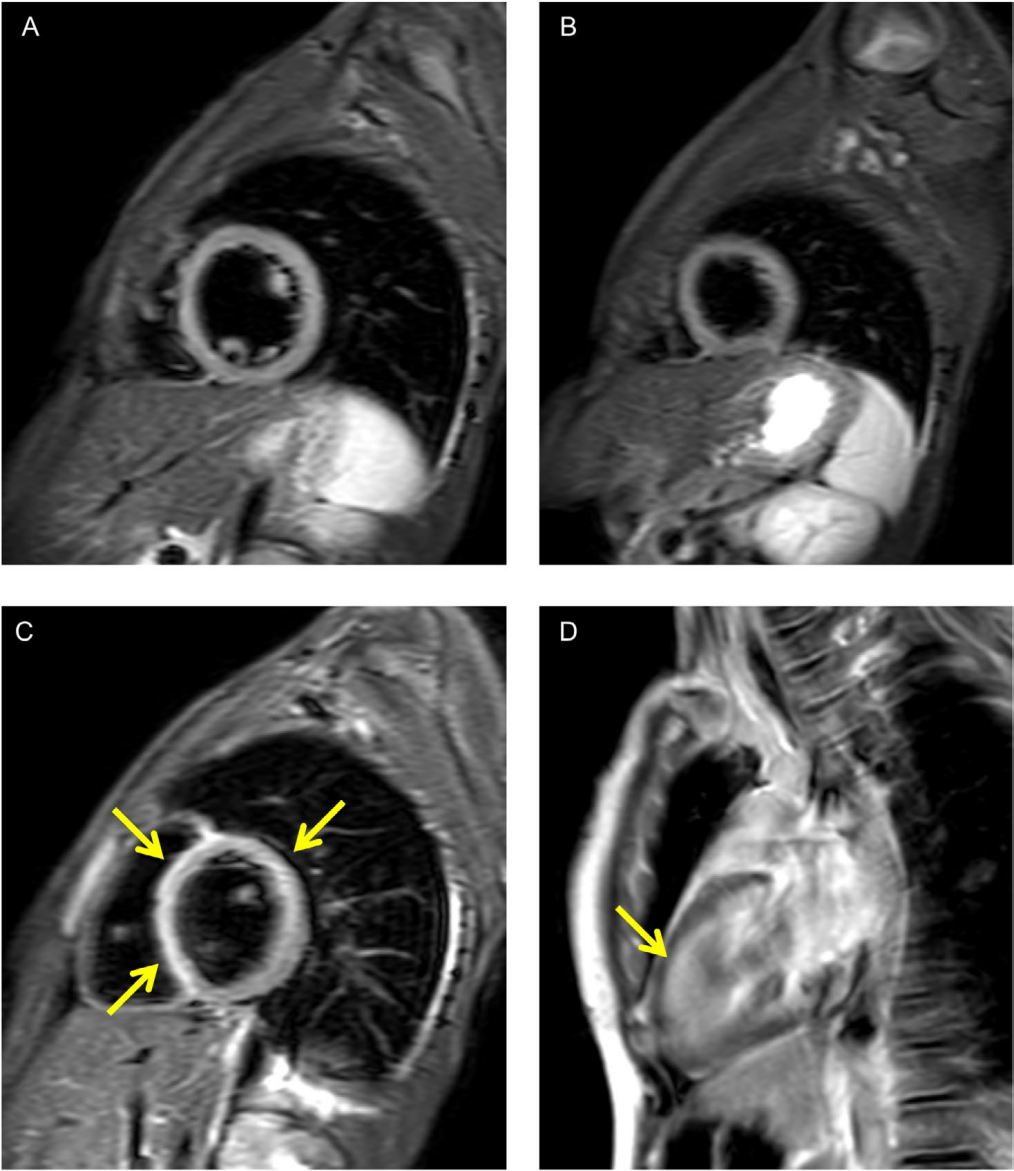
Cardiac magnetic resonance imaging in this patient. (A) Dark Blood T2 Short Inversion Time Reversal (STIR) image. No myocardial edema is observed. (B) Cine does not show an obvious localized wall motion abnormality. (C, D) Late gadolinium enhancement (LGE) imaging shows localized hyperintense signal within the myocardium. Yellow arrows indicate the enhancement.

The patient was a 12‐year‐old boy. He had no obvious perinatal abnormalities. Newborn screening showed low α‐galactosidase activity (4.4 U; cutoff < 17.0 U). Genetic testing in this patient identified the p.Gly43Asp variant in the *GLA* gene, resulting in a diagnosis of FD during infancy. This *GLA* variant is classified as the classical form of FD. His mother was confirmed to be heterozygous for the same variant as her son's. She had been experiencing acroparesthesia for some time. This boy had no other notable past medical history or medications. At age 6, as part of routine surveillance following the newborn screening diagnosis, his urine test showed the presence of mulberry bodies. Because mulberry bodies raised concern for organ involvement, particularly cardiac involvement given his classic phenotype, cardiac magnetic resonance imaging (MRI) was performed and revealed a localized hyperintense signal within the myocardium without myocardial edema and wall motion abnormalities (Figure 1a–d). Renal function was normal, with no proteinuria. Because his mother had been experiencing acroparesthesia, she hoped to undergo ERT at the same time as her son. After discussing treatment options with the patient's family, agalsidase alfa (0.2 mg/kg/biweekly) was selected for both the patient and his mother, in part because of its shorter infusion time. Based on his urinary and cardiac imaging findings, which indicated organ involvement despite the absence of symptoms, ERT was initiated at age 6.2 years. At the time of ERT initiation, he had no symptoms, and his globotriaosylsphingosine (Lyso‐Gb3) level was 76.4 nmol/L. At age 7.2 years, he began experiencing recurrent acroparesthesia, episodic pain in his extremities, triggered by exercise/infection, without angiokeratoma, hypohidrosis, or gastrointestinal symptoms.

## 3 Differential Diagnosis, Investigations and Treatment

**FIGURE 2:**
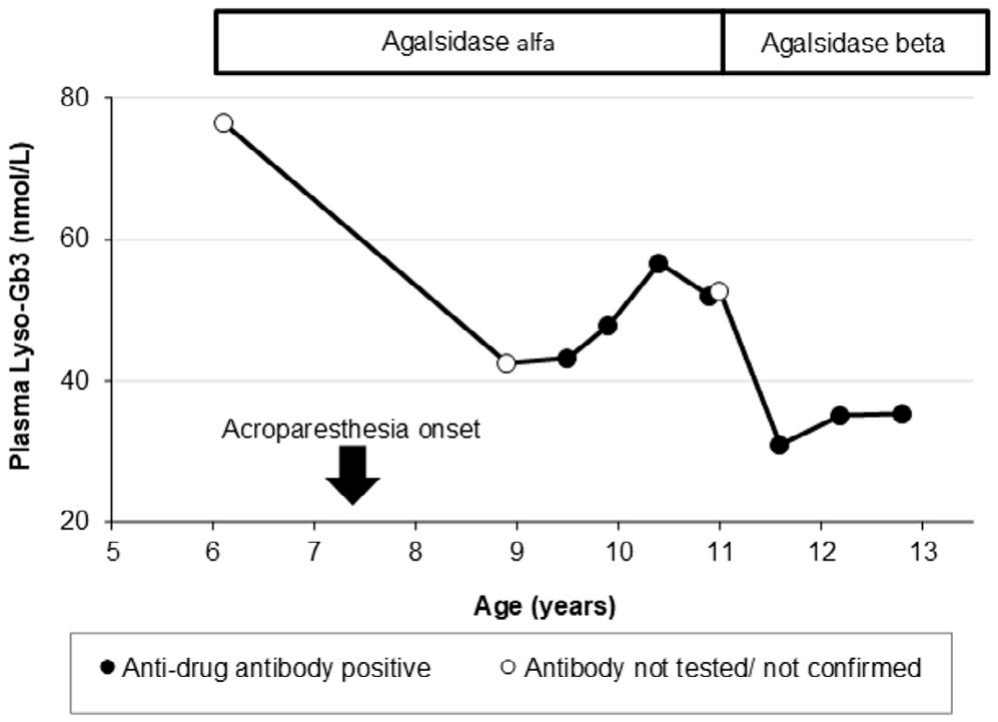
Clinical course of plasma Lyso‐Gb3 concentration before and after switching from agalsidase alfa to agalsidase beta. Plasma Lyso‐Gb3 concentrations are shown from age 6.2 to 12.8 years. This patient was treated with agalsidase alfa (age 6.2–10.9 years) and agalsidase beta (age 10.9 years onward). Filled circles indicate time points at which anti‐drug antibodies were confirmed positive; open circles indicate time points at which antibody testing was not performed or not confirmed. The arrow indicates the onset of acroparesthesia (age 7.2 years).

Given the genetically confirmed classic phenotype, trauma, infection, and rheumatic disease were considered unlikely, and the pain was attributed to FD. This pain was recurrent, seasonal, and lasted several days without fever, redness, swelling, or warmth, consistent with a Fabry‐related pain crisis. The pain was managed with as‐needed acetaminophen and typically resolved within several days, without affecting sleep or school attendance. Neuropathic pain‐specific agents such as pregabalin were not used, as this option was discussed with the patient and family and was not adopted in consideration of his young age, given the limited safety and dosing data for such agents in children with FD. The dose and frequency of agalsidase administration were not changed. We measured plasma Lyso‐Gb3 and anti‐drug antibody levels every 6 months. Despite continued agalsidase alfa therapy, the pain persisted, and plasma Lyso‐Gb3 remained elevated without a clear downward trend (Figure 2). Anti‐drug antibodies were confirmed at age 9.5 years; testing had not been performed at earlier time points. His Lyso‐Gb3 levels remained around 50.0 nmol/L (approximately a 35% reduction from pre‐treatment). Thus, we assumed the patient's pain was caused by Fabry disease. At age 10.9 years, we changed from agalsidase alfa to beta (1.0 mg/kg/biweekly) based on this insufficient clinical and biochemical response.

## 4 Outcome and Follow‐Up

After switching to agalsidase beta, his pain gradually improved. Recently, his acroparesthesia has improved within a day. We continued agalsidase beta without any change in dose or frequency. Anti‐drug antibodies were confirmed at age 11.6 years, approximately 9 months after the switch. During his latest visit, the Lyso‐Gb3 levels decreased to about 35.0 nmol/L (approximately a 54% reduction from pre‐treatment) despite a positive antibody to agalsidase beta. No abnormalities were found on echocardiogram, and renal function—including serum creatinine, eGFR, and urinary protein—was within normal limits.

## 5 Discussion

We described a patient with classical FD diagnosed through newborn screening. Notably, the diagnosis was made shortly after birth, well before the median age of symptom onset (approximately 6 years) or diagnosis (approximately 9 years) reported for boys with classic FD [5]. In Japan, a regional NBS study identified 26 hemizygous males, three of whom later developed symptoms and received ERT [17], indicating that ERT is already used in NBS‐identified pediatric patients in clinical practice. Although ERT was initiated while the patient remained asymptomatic, he subsequently began to experience acroparesthesia after starting agalsidase alfa, in the presence of anti‐drug antibodies. Additionally, switching ERT from agalsidase alfa to beta improved his acroparesthesia and decreased Lyso‐Gb3 levels. Our patient emphasizes that monitoring clinical symptoms, plasma Lyso‐Gb3 levels, and anti‐drug antibodies is important for managing patients with FD, even after initiating treatment. The pre‐treatment Lyso‐Gb3 levels in this patient were consistent with classic Fabry disease, as reported in previous studies [20]. The development of anti‐drug antibodies has been associated with reduced biochemical responses to the enzyme [21, 22], and has also been linked to poorer long‐term renal and cardiac outcomes in some studies [23]. Agalsidase alfa can relieve acroparesthesia and reduce classical patient plasma Lyso‐Gb3 by approximately 40%–50% [12, 24]. Thus, the reduction in Lyso‐Gb3 in our patient may have been insufficient. Furthermore, Lyso‐Gb3 levels in this patient decreased by more than 50% after switching to agalsidase beta, compared with pre‐treatment levels. These findings in the present study indicate that switching to the enzyme beta contributes to the relief of his extremity pain. It should be noted that this represents a switch in enzyme preparation rather than a dose increase within the same preparation, as agalsidase alfa and agalsidase beta are approved at substantially different standard doses (0.2 mg/kg and 1.0 mg/kg biweekly, respectively). Consistent with this, agalsidase beta allows high‐dose administration and maintains higher enzyme activity even in the presence of antibodies [21]. In this context, our findings suggest that switching to agalsidase beta may improve outcomes in patients with FD when the effect of agalsidase alfa is insufficient, given that Lyso‐Gb3 correlates with left ventricular mass [25]. However, plasma Lyso‐Gb3 levels vary considerably with the *GLA* variant, phenotype (classic vs. late‐onset), and sex; therefore, caution is warranted when interpreting treatment response based on this biomarker alone [20]. In addition, because acroparesthesia improves in a minority of FD patients [7], a natural course cannot be entirely excluded.

In this patient, early diagnosis through NBS contributed to early treatment. Our patient had abnormal urine test results before age 7. The cardiac MRI findings suggested mild cardiac involvement consistent with FD. Based on subclinical organ involvement, ERT with agalsidase alfa was initiated at age 6.2 years, while the patient remained asymptomatic. These findings suggest that an appropriate assessment of organ involvement is necessary during childhood, even when Fabry‐related manifestations are not obvious. Early initiation of ERT in asymptomatic pediatric patients has been reported to normalize disease biomarkers, supporting a proactive treatment approach [26]. In Japan, agalsidase alfa and agalsidase beta are approved for patients with a confirmed diagnosis of FD, with no lower age restriction. To date, treatment for classic males with FD has been recommended at symptom onset or around ages 7–10 [1], consistent with the European Fabry Working Group consensus [27]. Mulberry bodies have been reported to be linked to significant kidney involvement in previous studies [28, 29]. Similarly, a prior study indicated that mulberry bodies often prompt the initiation of treatment [29]. Hence, we assume that organ involvement had already progressed in this patient. Furthermore, the cardiac MRI showed abnormal lesions: A hyperintense signal on late gadolinium enhancement imaging might reflect myocardial fibrosis (Figure 1c,d). In this regard, Havranek et al. demonstrated that cardiac impairment can be detected in pediatric patients with FD and highlighted the importance of early cardiac evaluation in FD [30]. Consistent with this, previous findings indicate that globotriaosylceramide accumulation begins in the fetal period [31]. Further research is needed to assess cardiac abnormalities in childhood Fabry disease. In addition, because relief of acroparesthesia does not always correlate with improvement in organ dysfunction [6], we should keep in mind that long‐term follow‐up of pediatric patients detected by neonatal screening is important for selecting the optimal treatment for future pediatric cases of Fabry disease. In addition, the psychological and socioeconomic burden of very early diagnosis, and the long‐term efficacy of treatment started before symptom onset, remain subjects of ongoing debate [32]. As FD is a progressive, multi‐organ disease, regular follow‐up by a multidisciplinary team, including pediatrics, nephrology, cardiology, and neurology, is recommended [33]. Moreover, pegunigalsidase alfa, a newer agalsidase formulation, was not considered for this patient because it is not currently approved in Japan.

## 6 Conclusion

We outline the clinical course of a boy with classic FD diagnosed through newborn screening, in whom switching from agalsidase alfa to agalsidase beta was associated with a reduction in plasma Lyso‐Gb3 and an improvement in acroparesthesia. This report contributes to the pediatric experience with enzyme switching and broadens clinical understanding of FD in children identified through newborn screening.

## Author Contributions

**Nobuhiko Koga:** conceptualization, methodology, investigation, data curation, writing – original draft. **Hiroaki Yodogawa:** data curation. **Shinichiro Nagamitsu:** conceptualization, writing – review and editing. **Takahito Inoue:** conceptualization, methodology, investigation, writing – review and editing, supervision. **Yuichi Mushimoto:** data curation. **Takaaki Sawada:** conceptualization, writing – review and editing. **Kimitoshi Nakamura:** conceptualization. **Shinichi Hirose:** conceptualization.

## Funding

The authors have nothing to report.

## Consent

Written informed consent was obtained from the patient and parents.

## Conflicts of Interest

The authors declare no conflicts of interest.

## Data Availability

The data that support the findings of this study are available from the corresponding author upon reasonable request.
